# Effectiveness of Interdisciplinary Teaching on Creativity: A Quasi-Experimental Study

**DOI:** 10.3390/ijerph19105875

**Published:** 2022-05-12

**Authors:** Hsing-Yuan Liu, Ding-Yang Hsu, Hui-Mei Han, I-Teng Wang, Nai-Hung Chen, Chin-Yen Han, Sheau-Ming Wu, Hsiu-Fang Chen, Ding-Hau Huang

**Affiliations:** 1 Department of Nursing, Chang Gung University of Science and Technology, No. 261, Wunhua 1st Rd., Gueishan Township, Taoyuan City 33303, Taiwan; itwang@mail.cgust.edu.tw (I.-T.W.); nhchen@mail.cgust.edu.tw (N.-H.C.); cyhan@mail.cgust.edu.tw (C.-Y.H.); sheamwu@mail.cgust.edu.tw (S.-M.W.); fang@mail.cgust.edu.tw (H.-F.C.); 2 Research Fellow (Joint Appointment), Linkou Chang Gung Memorial Hospital, No. 5, Fuxing St., Guishan Dist., Taoyuan City 333423, Taiwan; 3 Department of Industrial Design, Ming Chi University of Tchnology, 84 Gungjuan Rd., Taishan Dist., New Taipei City 243303, Taiwan; hsuchiapin@mail.mcut.edu.tw; 4 Department of Nursing, Linkou Chang Gung Memorial Hospital, No. 5, Fuxing St., Guishan Dist., Taoyuan City 333423, Taiwan; t22026@cgmh.org.tw; 5 Department of Finance, National United University, No. 1, Lienda, Miaoli 360301, Taiwan; 6 Institute of Creative Design and Management, National Taipei University of Business, No. 100, Sec. 1, Fulong Rd., Pingzhen Dist., Taoyuan City 324022, Taiwan; hauhuang@ntub.edu.tw

**Keywords:** divergent thinking abilities, interdisciplinary teaching, nursing student teams, team creativity

## Abstract

Little is known about the effectiveness of Interdisciplinary teaching (IDT) in higher education, particularly for healthcare education in Taiwan. It is vital to determine if IDT could enhance divergent creative thinking and team creativity among nursing students. A quasi-experimental study with a pretest-posttest design. Students enrolled in a capstone nursing course for the development of healthcare-related products were divided into two groups. The intervention group (*n* = 61) was taught creative thinking skills with IDT by faculty. The control group (*n* = 84) was taught by nursing faculty with traditional teaching. This study found that students who received the IDT intervention scored significantly higher on measures of creative thinking and team creativity compared with students in the control group. These findings suggest integrating IDT from nursing and design faculty into the teaching curriculum to foster students’ creative thinking abilities when formulating interdisciplinary student teams to develop innovative, creative healthcare products.

## 1. Introduction

Interprofessional Education (IPE) is a collaboration of healthcare professionals across multiple disciplines, which can improve the quality of patient care [1]. Information technology (IT) has recently become a fundamental element of healthcare as the volume of medical records increases, which includes a patient’s history, test results, and X-ray exams over the lifetime of the patient [2]. The design of innovative healthcare products has also increased the impact of science and engineering on technology as well as patient care, especially as it pertains to nursing education [3]. Nursing programs that offer IPE to encourage students to contribute to the invention of innovative healthcare products offer nursing students an opportunity to collaborate with students in other disciplines, such as engineering or design. IPE courses are designed to improve students’ attitudes, knowledge, skills, and behaviors during collaborative activities [4]. According to Woermann et al. [5], capstone courses in nursing programs comprised of IPE teams with students from non-nursing backgrounds, whose goal is to create practical healthcare technology, may, therefore, better prepare nursing students to collaborate creatively and use technology more effectively as members of interdisciplinary healthcare teams trained to deliver safe, comprehensive patient care. In healthcare education, the IPE model teaches healthcare students to work closely with students of other disciplines as preparation for engaging effectively with the diverse teams of healthcare professionals they will encounter in their careers [6]. By training nursing students to effectively communicate and collaborate on teams with students from other backgrounds, the IPE model prepares future nurses to provide safer, higher quality patient care [6]. The Association for Medical Education in Europe encourages the use of IPE for all students in the medical sciences [7], and healthcare curricula in Taiwan began incorporating IPE into capstone courses in 2016 [8]. However, only a limited number of universities offer nurses an opportunity to pursue an education that includes exposure to interdisciplinary fields [2].

Improving interprofessional collaboration in healthcare has been recognized internationally as important for improving patient outcomes [9,10,11]. However, there is a paucity of research on quantitative assessments to determine whether the collaboration among disciplines facilitated by IPE is effective in healthcare [12]. The concept of interprofessional collaboration has been encouraged in clinical care in Taiwan since 2004, however, it was not until 2016 that Taiwan adopted IPE as a formal part of the teaching curriculum for clinical healthcare education [8,9].

Interdisciplinary teaching (IDT) is a strategy used in academic programs that include IPE because it incorporates principles from multiple disciplines in order to provide a richer understanding of a topic. Liu [13] indicated that IDT is a collaboration of healthcare professionals across multiple areas, which can enhance the quality of patient care [1]. The complex nature of healthcare systems involves components that differ yet are interconnected [14]. Thus, providing education from nursing faculty with a broad range of knowledge and an understanding of the complexity of the healthcare system could allow nurses to have a better grasp of the technological and methodological challenges of patient care [3]. The importance of facilitating IDT in higher education in the sciences and medicine has been recognized by several universities worldwide including the United States, Germany, and Japan [15]. However, in Taiwan, IDT at the level of higher education is in the developmental stage, particularly for healthcare education. Therefore, the effectiveness of IDT in higher education in Taiwan needs further exploration.

Although studies have investigated IDT, there is no consensus on how the effectiveness of this teaching format should be evaluated [16,17,18,19,20]. Creativity and innovation are considered important core competencies for nursing students in Taiwan [21]. While there has been an increasing interest in interdisciplinary teaching in Taiwan, there is a handful of empirical evidence to support the effectiveness of this process [21,22]. Additionally, creativity has been shown to be increased through training [21,22,23,24]. Whether a creativity training program is effective requires a valid assessment of creativity [21,25].

The Torrance Tests of Creative Thinking (TTCT) are a valid predictor of creative achievement and scores are highly related to divergent thinking scores [26,27,28]. However, only a few studies have examined the effectiveness of creativity training programs focused on higher education [29] or nursing education [21,22], especially for nursing students in Taiwan, and whether it can help them work collaboratively.

## 2. Materials and Methods

### 2.1. Objective of Research

The objective of this research was to evaluate whether an Interdisciplinary Teaching (IDT) can enhance divergent creative thinking and team creativity for Interprofessional Education (IPE) capstone course for nursing students.

### 2.2. Research Design, Participants, and Procedure

A quasi-experimental study with a pretest-posttest design was comprised of a non-equivalent intervention group and a control group of nursing students. Nursing students enrolled in an IPE capstone course were recruited from a university of science and technology in northern Taiwan. The 18-week capstone course is part of a 2- and 4-year nursing program. The course is focused on applying design thinking to define a healthcare problem, generate ideas for products that could solve the problem, and create and test a prototype of a patentable healthcare-related product.

**Figure 1:**
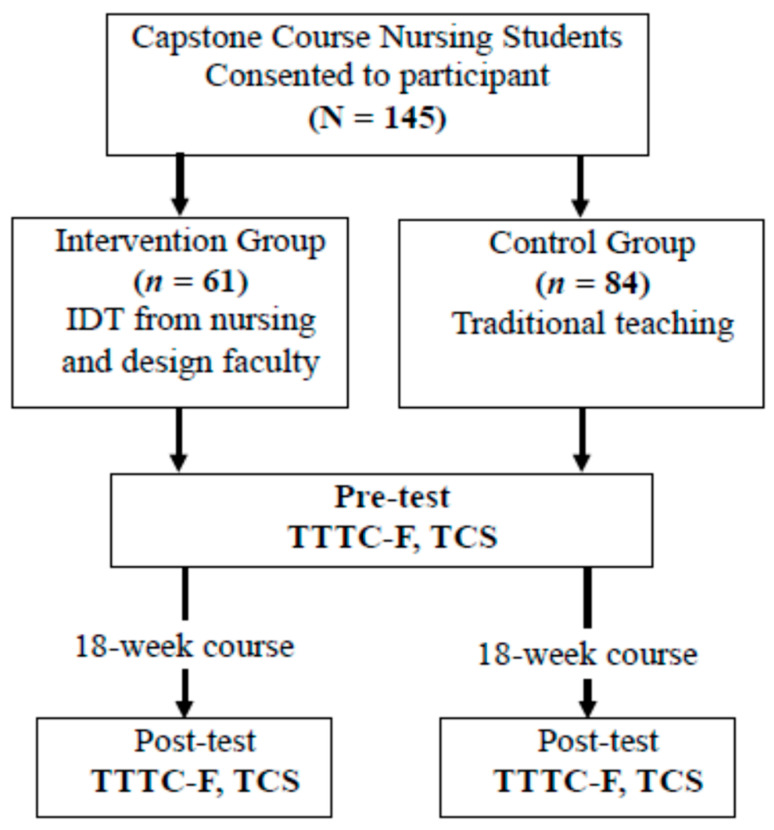
Flowchart of interdisciplinary teaching intervention in teaching creativity and the control group.

We used G*Power to calculate the required minimum sample size and used F-test (ANOVA), with effect size set to 0.25 (medium effect), confidence level set at 95%, power as 0.8, and *n* = 2 for groups. The minimum sample size required was determined to be 128 [30]. Information packets about the program were provided to 177 nursing students, and 145 agreed to participate and provided written informed consent. Students were unaware of any difference in faculty teaching the course at the time of enrollment. The 145 nursing students were divided into two groups: The intervention group (*n* = 61) was taught by interdisciplinary faculty from departments of nursing and design. Students enrolled in the course were from the nursing program and a design program from another university. The control group (*n* = 84) received traditional teaching from the nursing faculty. Participants were given a coded packet containing paper-and-pencil questionnaires to be filled out on the first day of the semester and after completion of the course at the end of the semester. The flowchart of the study is shown in Figure 1.

Data were collected between September 2019 and January 2020 from survey questionnaires. One questionnaire was used to obtain data regarding participants’ demographics (age, gender). Two self-report questionnaires were used to measure divergent creative thinking and team creativity, as described below.

### 2.3. The Intervention Program

Students in the IDT intervention group met together for three 4-h workshops. However, most collaboration and communication occurred electronically via web conferencing and instant messaging services. The first workshop included helping nursing and design students understand each other’s specialty; instruction in team collaboration; how to create online groups for collaborations to discuss weekly tasks and homework updates; and faculty’s expectations about group presentations of a novel healthcare product and faculty evaluations. The second workshop involved a visit to a design factory and instruction in prototyping. During the midterm exam, final workshop, and term exam, students presented their products to three experts of varying backgrounds (clinical nursing, medical engineering, and industrial design) who provided feedback to the students about the design of their prototype. Students in the control group met together in the regular classroom and received traditional teaching during the classes. They have instructed the same curriculum structure as students in the intervention group such as three 4-h workshops, and midterm and final presentations. The major difference between students in the control and intervention groups is that students in the control group were instructed by nursing teachers and their teams were formulated by nursing students. An overview of the intervention is shown in Table 1.

### 2.4. Instruments

Divergent creative thinking was assessed in the Taiwanese version of the Torrance Tests of Creative Thinking (TTCT) developed by Wu [31]. The Taiwanese version of the TTCT is based on the TTCT scale of Torrance [32] and was developed to assess the divergent creative thinking ability in Taiwan. The TTCT has 2 parts: a verbal version, Form B (TTCT-V), and a figural version, Form A (TTCT-F). In this study, we used the TTCT-F to measure students’ divergent creative thinking abilities since the TTCT-F assessment is a more comprehensive, reliable, and valid measure of creativity than the TTCT-V 17,22. The TTCT-F is comprised of four constructs including fluency (number of relevant responses), flexibility (number of different categories of relevant responses), originality (statistical rarity of the responses), and elaboration (amount of detail in the responses). The range for the total score of the TTCT-F is 0–377. The ranges for the subscale scores of fluencies, flexibility, originality, and elaboration are 0–37, 0–35, 0–114, and 0–171, respectively. Cronbach’s alpha coefficients were computed to determine the reliability estimates of the TTCT for this study. In this study, the Cronbach alpha coefficient for the total score for the TTCT-F was 0.82 and satisfactory validity was established for this version through factor analyses.

We measured how the nursing students perceived their team’s creativity with the 10-item team creativity scale (TCS) developed by Yang, Xie, & Bao [33]. The scale is based on the team creativity scale of Farh, Lee, and Farh and was developed to assess the creativity of teams in China [34]. The 10 items are statements about team members’ behavior during collaborative activities, such as “team members produce products that are creative”, or “team members frequently propose suggestions for different solutions by sharing ideas”. Items are scored on a 5-point Likert scale ranging from 1 (strongly agree) to 5 (strongly disagree) and the total score is the average of the sum of the 10 items. Scores range from 1–5; higher scores indicate higher perceived levels of team creativity. The scale has a Cronbach’s alpha of 0.95 indicating good reliability and factor analysis established satisfactory validity for the scale [11,33]. In this study, Cronbach’s alpha was 0.95. Table 2 contains example statements for the TCS.

### 2.5. Data Analysis

After all packets were collected, data were entered into a computer and analyzed using SPSS version 20.0. Descriptive statistics using the mean and standard deviation (SD) evaluated the characteristics of the participants and scores on the self-report questionnaires. Analysis of covariance (ANCOVA) determined if the mean post-test scores for the control and intervention groups differed. ANCOVA is useful for determining if post-test means, adjusted for mean pre-test scores, differ between two groups [35].

### 2.6. Ethical Considerations

The study was approved by the Institutional Review Board (IRB) of the hospital ethics committees (IRB numbers: 201800212B0) prior to data collection. All data were collected anonymously. The director of the healthcare program checked for the presence of a signed consent form and removed it from the packet to ensure the confidentiality of the students’ information. Only packets containing a signed written informed consent form were included in the data analysis.

## 3. Results

### 3.1. Sample Characteristics and Mean Scale Scores for TTCT-F and TCS

All 145 nursing students were female and the mean age was 21.5 years (SD = 1.19). There was no significant difference in age between students in the intervention and control groups. Pre-test and post-test scores for the TTCT-F and TCS are shown in Table 3. There was no significant difference in pre-test scores between the intervention and control groups. The total mean scores for all students for the TTCT-F and TCS were 40.46 (SD = 13.35) and 38.26 (SD = 6.08), respectively. The highest mean subscale score of the TTCT-F was for fluency (M = 19.01, SD = 6.26) and the lowest was elaboration (M = 1.50, SD = 1.93). The intervention group had significantly higher mean post-test scores than the control group for total and subscales scores on the TTCT-F and total scores on the TCS.

### 3.2. ANCOVA for the Control and Intervention Group

Differences between the control and intervention groups on post-test scores for teaching behaviors and teaching creativity were compared using one-way ANCOVA, controlling for pre-test scores are shown in Table 4. There was a significant effect of group type on post-test scores for the TTCT-F (*p* < 0.001) and the subscales of fluency (*p* < 0.001), originality (*p* = 0.003), and elaboration (*p* < 0.001). Total post-test scores were also significantly different for the TCS (*p* = 0.02).

## 4. Discussion

This study investigated the effects of an IDT intervention provided by nursing and design faculty on creative thinking abilities and team creativity among nursing student teams in Taiwan. Nursing students who participated in the IDT intervention program improved scores on the TTCT-F and TCS compared with students in the control group. These findings confirm our hypothesis that the IDT intervention program had a positive effect on both creative thinking abilities and team creativity.

Our findings reinforce those of previous studies on the effectiveness of creativity training programs on students’ creative thinking abilities [23,24]. These findings contrast with those of Chan, Huang, and Wu who reported a creative thinking course did not improve figural creative thinking abilities for students in home economics [36]. A possible explanation for this is that nursing students in this study collaborated with design students and their collaborative interactions stimulated their creative abilities. Our findings are also consistent with previous studies that showed creativity training improved Taiwanese nursing students’ self-perceived abilities of creativity [21,37]. However, the sample size in the study by Ku et al. [37] was too small to demonstrate a significant improvement.

All post-test subscale scores for the TTCT-F also increased for students in the IDT intervention program. This finding contrasts with the study by Liu et al. [31] who reported the TTCT-F subscale score for elaboration did not improve for nursing students who received IDT from faculty with creativity training. One possible explanation could be that the intervention program integrated design thinking tools as a component of the creative teaching strategy and fostered nursing students’ elaboration of creative thinking abilities. The increase seen in our study across all subscales of the TTCT-F may be due to the structure of the workshops and the additional component of online group discussions which may have strengthened students’ creative skills.

This is the first study to report a significant improvement in team creativity for nursing student teams following training in design thinking and creativity. The significant increase in mean post-test scores on the TCS suggests the IDT intervention had a positive impact on nursing students’ perception of their team’s creativity. Instruction not only in design thinking and creativity but also the ongoing collaboration that the online groups allowed, may have increased the ability of the student teams to develop a more positive perspective of the team’s creative abilities. Our findings regarding the effectiveness of IDT and exposure to creativity skills on team creativity expand on previous findings showing significant improvement in student interdisciplinary thinking, interprofessional collaboration skills, and creative process skills following an IDT program [16,17,18].

The findings have some limitations. First, our quasi-experimental study employed a nonrandom design, which may not control for all factors that influence the inner validity of the experiment. On the other hand, a quasi-experimental study with a non-random design prevents concluding causal associations between the intervention and outcomes. In addition, the IDT was a short-term intervention. Therefore, a longitudinal study such as a generalized estimating equation (GEE) analysis is recommended for future studies [13]. Second, we only assessed students from one university, which limits the applicability of our findings, and the lack of male participants limits the generalization of our findings. Therefore, future studies should include participants from other nations with varied cultural and gender diversity. future research in this area would be strengthened by assessing team interdisciplinarity from the perspective of non-nursing students who participate in IPE nursing program capstone courses [13,38]. Finally, our analysis of team creativity was derived from students’ self-reported perceptions and may not provide an objective reflection of team creativity. Therefore, we recommend that future studies include objective measurements, such as nursing students’ grades or faculty-scored standardized creativity assessments.

## 5. Conclusions

This study explored the effects of IDT intervention delivered by nursing and design faculty on creative thinking abilities and team creativity among nursing students in Taiwan. Our findings indicated that the mean total score of 40.46 (SD = 13.35) on the Taiwanese version of the TTCT-F is lower than reported for college students [39] and 50% lower than nursing in the United States [40]. Liu et al. [41] reported similar results. One similarity between the nursing students in our study and the students in the Washburn University study scored the lowest on the construct of elaboration. This suggests more emphasis should be placed on incorporating techniques such as mind-mapping in order to improve the ability to elaborate on an idea.

The IDT intervention program was designed to nurture and enhance creative thinking abilities among nursing students. Our findings are the first to demonstrate that providing nursing student teams instruction in creativity using IDT can increase students’ creative thinking abilities and team creativity. The implications of our findings are especially important for nursing education in Taiwan due to the current emphasis on improving creativity and innovation for nursing students in Taiwan. We suggest integrating IDT from nursing and design faculty into the teaching curriculum to foster students’ creative thinking abilities when formulating interdisciplinary student teams to develop innovative, creative healthcare products.

## Figures and Tables

**Table 1:** The Interdisciplinary Teaching Intervention Program: The three 4-h workshops, and content of the 18-week capstone course.

Section	Time	Content
**Workshop I**	4 h	
1. Introduction		Guidance on understanding each other’s specialty
2.Team collaboration		Instruction in how to effectively collaborate with others during presentations of creative healthcare products Instruction in how to create online groups for discussing weekly homework tasks and updates.

3. Design thinking approaches		Introduction to design thinking and case studies of inventions of healthcare products
		Instruction in guiding small groups of students to define problems from a viewpoint of empathy
		Group presentations and feedback from nursing and design faculty
**Interdisciplinary online group discussions** Design thinking approaches	14 h	Discuss how to observe and define problems from a design-thinking perspective. Use of brainstorming verify the results and effectiveness of different solutions Use of brainstorming to find the most appropriate solution
	
**Workshop II (advanced)**	4 h	
1. Visit a design factory		See how a variety of prototyping is used to implement designs (e.g., 3D printing building blocks/poster/cardboard)
2. Use of design thinking approach: Prototyping		Communicate with users through the visualization of prototypes. Use event prototyping to assess needs early and correct feasible solutions
3. Midterm presentation		Group presentation with feedback from three experts
**Group work:**	10 h	
1. Do product and test 2. Use of design thinking approach		Group implementation of health-related product Products applicability assessment through testing

**Table 2:** Example Statements of Items for the Team Creativity Scale.

Item	During the Capstone Team Activities, Team Members:
1	Produce products that are creative.
2	Use new methods to complete tasks.
3	Find new uses for resources, knowledge, and skills.
4	Frequently propose suggestions for different solutions by sharing ideas.
5	Adopt new methods to resolve questions, even when they expect the methods might be difficult to execute.

**Table 3:** Characteristics, Mean Scale, and Subscale Scores for Nursing Students in the Control (*n* = 84) and Intervention Group (*n* = 61).

Pre-Test Scores				Post-Test Scores	
	All	Control	Intervention	Control	Intervention
Characteristics/Scale	M (SD)	M (SD)	M (SD)	M (SD)	M (SD)
Age (year) TTCT-F	21.50 (1.19)	21.56 (1.50)	21.34 (0.51)	21.56 (1.50)	21.34 (0.51)
Total (range = 0–377)	40.46 (13.35)	39.89 (13.34)	41.23 (13.43)	42.82 (20.41)	53.86 (15.54)
Subscale					
Fluency (range = 0–37)	19.01 (6.26)	18.96 (6.67)	19.07 (5.70)	18.24 (8.78)	22.69 (6.81)
Flexibility (range = 0–35)	10.44 (2.78)	10.20 (2.85)	10.77 (2.67)	11.11 (4.46)	12.70 (2.61)
Originality (range = 0–114)	9.51 (5.05)	9.29 (4.79)	9.82 (5.41)	11.89 (7.98)	16.11 (7.55)
Elaboration (range = 0–171)	1.50 (1.93)	1.44 (1.11)	1.57 (2.68)	1.58 (1.15)	2.36 (1.39)
TCS					
Total (range = 0–50)	38.26 (6.08)	39.51 (6.48)	37.35 (6.37)	38.73 (7.35)	42.13 (5.52)

**Table 4:** ANCOVA of adjusted Post-test scores on the TTCT-F and TCS for the Intervention (*n* = 61) and Control (*n* = 84) Groups.

	Intervention	Control					
Construct	Post-Test Mean	Post-Test Mean	SS	df	MS	F	*p*
TTCT-F							
Total score	51.73	42.14	3245.92	1	3245.92	10.73	0.001
Subscales							
Fluency	21.99	17.80	621.21	1	621.21	11.83	0.001
Flexibility	12.71	11.11	86.78	1	86.78	5.00	0.027
Originality	15.48	11.77	486.32	1	486.32	9.12	0.003
Elaboration	2.29	1.54	20.‘1	1	20.12	11.86	0.001
TCS (total score)	41.56	39.14	201.35	1	201.35	5.45	0.02

## Data Availability

The data that support the findings of this study are available on request from the corresponding author, H.-Y.L.

## References

[1] Birk J.T. (2017). Principles for developing an interprofessional education curriculum in a healthcare program. J. Healthc. Commun..

[2] Kim H.N. (2019). A conceptual framework for interdisciplinary education in engineering and nursing health informatics. Nurse Educ. Today.

[3] Davis C.R., Glasgow M.E.S. (2017). Nurse-scientists and nurse-engineers. Am. Nurse Today.

[4] Barr H., Koppel I., Reeves S., Hammick M., Freeth D.S. (2008). Effective Interprofessional Education: Argument, Assumption and Evidence (Promoting Partnership for Health).

[5] Woermann U., Weltsch L., Kunz A., Stricker D., Guttormsen S. (2016). Attitude towards and readiness for interprofessional education in medical and nursing students of Bern. GMS J. Med. Educ..

[6] Liu H.Y., Wang I.T., Hsu D.Y., Huang D.H., Chen N.H., Han C.Y., Han H.M. (2020). Conflict and interactions on interdisciplinary nursing student teams: The moderating effects of spontaneous communication. Nurse Educ. Today.

[7] Hean S., Craddock D., Hammic M., Hammic M. (2012). Theoretical insights into interprofessional education: AMEE Guide No. 62. Med. Teach..

[8] Yeh H.C., Huang S.Y., Chen T.Y., Hsieh M.C. (2019). An objective structured teaching exercise for faculty training and assessment of teaching ability in interprofessional collaborative practice and education. Tzu-Chi Med. J..

[9] Bosch B., Mansell H. (2015). Interprofessional collaboration in health care: Lessons to be learned from competitive sports. Can. Pharm. J. Rev. Des Pharm. Du Can..

[10] World Health Organization (2010). Framework for Action on Interprofessional Education and Collaborative Practice.

[11] Liu H.Y. (2020). Inter-professional nursing education and the roles of swift trust, interaction behaviors, and creativity: A cross-sectional questionnaire survey. Nurse Educ. Today.

[12] Guraya S.Y., Barr H. (2018). The effectiveness of interprofessional education in healthcare: A systematic review and meta-analysis. Kaohsiung J. Med. Sci..

[13] Liu H.Y. (2021). Effect of interdisciplinary teaching on collaborative interactions among nursing student teams in Taiwan: A quasi-experimental study. Nurse Educ. Today.

[14] Lipsitz L.A. (2012). Understanding health care as a complex system: The foundation for unintended consequences. JAMA.

[15] Li Q. (2020). Interdisciplinary Education in Language Universities–A Survey on Postgraduate Students’ Attitude and Its Implications. High. Educ. Stud..

[16] Dallaire L.F., Rhéaume C., Vézina L. (2018). Interdisciplinary teaching in family medicine teaching units: The residents’ points of view. Can. Med. Educ. J..

[17] Klaassen R., De Fouw N., Rooij R., van der Tang Y. Perceptions of Interdisciplinary Learning: A qualitative approach. Proceedings of the 8th Research in Engineering Education Symposium, REES 2019-Making Connections. Research in Engineering Education Network.

[18] Spelt E.J.H., Luning P.A., van Boekel M.A., Mulder M. (2017). A multidimensional approach to examine student interdisciplinary learning in science and engineering in higher education. Eur. J. Eng. Educ..

[19] Daly S.R., Mosyjowski E.A., Seifert C.M. (2019). Teaching creative process across disciplines. J. Creat. Behav..

[20] Bishop-Williams K.E., Roke K., Aspenlieder E., Troop M. (2017). Graduate Student Perspectives of Interdisciplinary and Disciplinary Programming for Teaching Development. Can. J. Scholarsh. Teach. Learn..

[21] Liu H.Y., Wang I.T. (2019). Creative teaching behaviors of health care school teachers in Taiwan: Mediating and moderating effects. BMC Med. Educ..

[22] Liu H.Y., Wang I.T., Chen N.H., Chao C.Y. (2020). Effect of creativity training on teaching for creativity for nursing faculty in Taiwan: A quasi-experimental study. Nurse Educ. Today.

[23] Azevedo I., de Fátima Morais M., Martins F. (2019). The future problem solving program international: An intervention to promote creative skills in Portuguese adolescents. J. Creat. Behav..

[24] Ritter S., Mostert N. (2016). Enhancement of creative thinking skills using a cognitive based creativity training. J. Cogn. Enhanc..

[25] Puccio G.J., Murdock C.M. (1999). Creativity Assessment: Readings and Resources.

[26] Torrance E.P. (1963). Creative Motivation Scale: Norms Technical Manual.

[27] Kim K.H. (2017). The Torrance tests of creative thinking-figural or verbal: Which one should we use?. Creat. Theor. Res. Appl..

[28] Kim K.H. (2008). Meta-analyses of the relationship of creative achievement to both IQ and divergent thinking test scores. J. Creat. Behav..

[29] Puccio G.J., Burnett C., Acar S., Yudess J.A., Holinger M., Cabra J.F. (2020). Creative problem solving in small groups: The effects of creativity training on idea generation, solution creativity, and leadership effectiveness. J. Creat. Behav..

[30] Faul F., Erdfelder E., Lang A.G., Buchner A. (2007). G* Power 3: A flexible statistical power analysis program for the social, behavioral, and biomedical sciences. Behav. Res. Methods.

[31] Wu C.S. (2002). The important concepts and implementation strategies of creative teaching. Taiwan Educ. Rev..

[32] Torrance E.P. (1995). Why Fly? A Philosophy of Creativity.

[33] Yang Z.R., Xie Z.S., Bao G.M. (2010). The mechanism of teams’ swift trust and interaction behavior on team creativity. J. Fuzhou Univ. (Philos. Soc. Sci.).

[34] Farh J.L., Lee C., Farh C.I. (2010). Task conflict and team creativity: A question of how much and when. J. Appl. Psychol..

[35] Brown M.B., Forsythe A.B. (1974). Robust tests for the equality of variances. J. Am. Stat. Assoc..

[36] Chan C.H., Huang H.H., Wu M.H. (2009). A study of the teaching effectiveness of creative thinking in a home economics course. J. Technol. Vocat. Educ..

[37] Ku Y.L., Lee P.Y., Shen M.H., Kuo C.L. (2014). Constructing and evaluating a nursing capstone course for cultivating creativity in RN-BSN students in Taiwan. J. Nurs. Educ. Pract..

[38] Liu H.Y. (2022). Promoting creativity of nursing students in different teaching and learning settings: A quasi-experimental study. Nurse Educ. Today.

[39] Karpova E., Marcketti S.B., Barker J. (2011). The efficacy of teaching creativity: Assessment of student creative thinking before and after exercises. Cloth. Text. Res. J..

[40] Washburn University, Office of Academic Affairs (2019). 2017–2018 Torrance Test of Creative Thinking Results.

[41] Liu H.Y., Wang I.T., Huang D.H., Hsu D.Y., Han H.M. (2020). Nurturing and enhancing creativity of nursing students in Taiwan: A quasi-experimental study. J. Creat. Behav..

